# Operando X-ray absorption spectra and mass spectrometry data during hydrogenation of ethylene over palladium nanoparticles

**DOI:** 10.1016/j.dib.2019.103954

**Published:** 2019-04-26

**Authors:** Aram L. Bugaev, Alexander A. Guda, Ilia A. Pankin, Elena Groppo, Riccardo Pellegrini, Alessandro Longo, Alexander V. Soldatov, Carlo Lamberti

**Affiliations:** a The Smart Materials Research Institute, Southern Federal University, Sladkova 178/24, 344090 Rostov-on-Don, Russia; b Department of Chemistry, INSTM and NIS Centre, University of Turin, Via Quarello 15, 10125 Turin, Italy; c Chimet SpA - Catalyst Division, Via di Pescaiola 74, Viciomaggio Arezzo, 52041 Italy; d Netherlands Organization for Scientific Research at ESRF, BP 220, F-38043 Grenoble Cedex 9, France; e Istituto per Lo Studio Dei Materiali Nanostrutturati (ISMN)-CNR, UOS Via Ugo La Malfa, 153, 90146 Palermo, Italy; f Department of Physics and CrisDi Interdepartmental Centre, University of Turin, Via P. Giuria 1, 10125 Turin, Italy

## Abstract

We report the series of Pd *K*-edge X-ray absorption spectra collected during hydrogenation of ethylene with variable ethylene/hydrogen ratio over carbon supported palladium nanoparticles. The data presented in this article includes normalized X-ray absorption spectra, *k*^2^-weighted oscillatory χ(*k*) functions extracted from the extended X-ray absorption fine structure (EXAFS) and *k*^2^-weighted Fourier-transformed EXAFS data, χ(*R*). Each spectrum is reported together with the hydrogen, ethylene and helium flow rates, adjusted during its collection. In addition, time evolution of the ratio of *m*/*Z* signals of 30 and 28 registered by online mass spectrometer is presented. The data analysis is reported in Bugaev et al., *Catal. Today*, 2019 [1].

**Table:** Specifications table

Subject area	*Catalysis, spectroscopy, nanomaterials*
More specific subject area	*Operando spectroscopy of palladium nanocatalyst*
Type of data	*Table, text file, figure*
How data was acquired	*X-ray absorption spectra were collected in transmission mode at BM26A beamline of ESRF synchrotron. Mass spectrometry data were collected by online mass spectrometer manufactured by Pfeiffer.*
Data format	*Processed, Analyzed*
Experimental factors	*X-ray absorption spectra in transmission mode*
Experimental features	*Carbon supported palladium nanoparticles pre-reduced in hydrogen exposed to the reaction mixture with variable ethylene/hydrogen ratio*
Data source location	*Grenoble, France (45.209749, 5.688410)*
Data accessibility	*Data is provided with this article*
Related research article	*Companion paper to: A. Bugaev* et al. *The Role of Palladium Carbides in the Catalytic Hydrogenation of Ethylene over Supported Palladium Nanoparticles. Catal. Today, in press,*https://doi.org/10.1016/j.cattod.2019.02.068*.*

**Table:** 

**Value of the data** • The dataset of X-ray absorption spectra is measured on well-defined Pd nanocatalyst during ethylene hydrogenation. • The EXAFS data can be re-used within single- and multiple-shell Fourier analysis. • The XANES data of well-defined 2.6 nm palladium nanoparticles can be exploited for extension of a machine learning databases. • Deep analysis of X-ray absorption spectra may provide an insight on catalyst degradation under working conditions.

## 1 Data

**Fig. 1:**
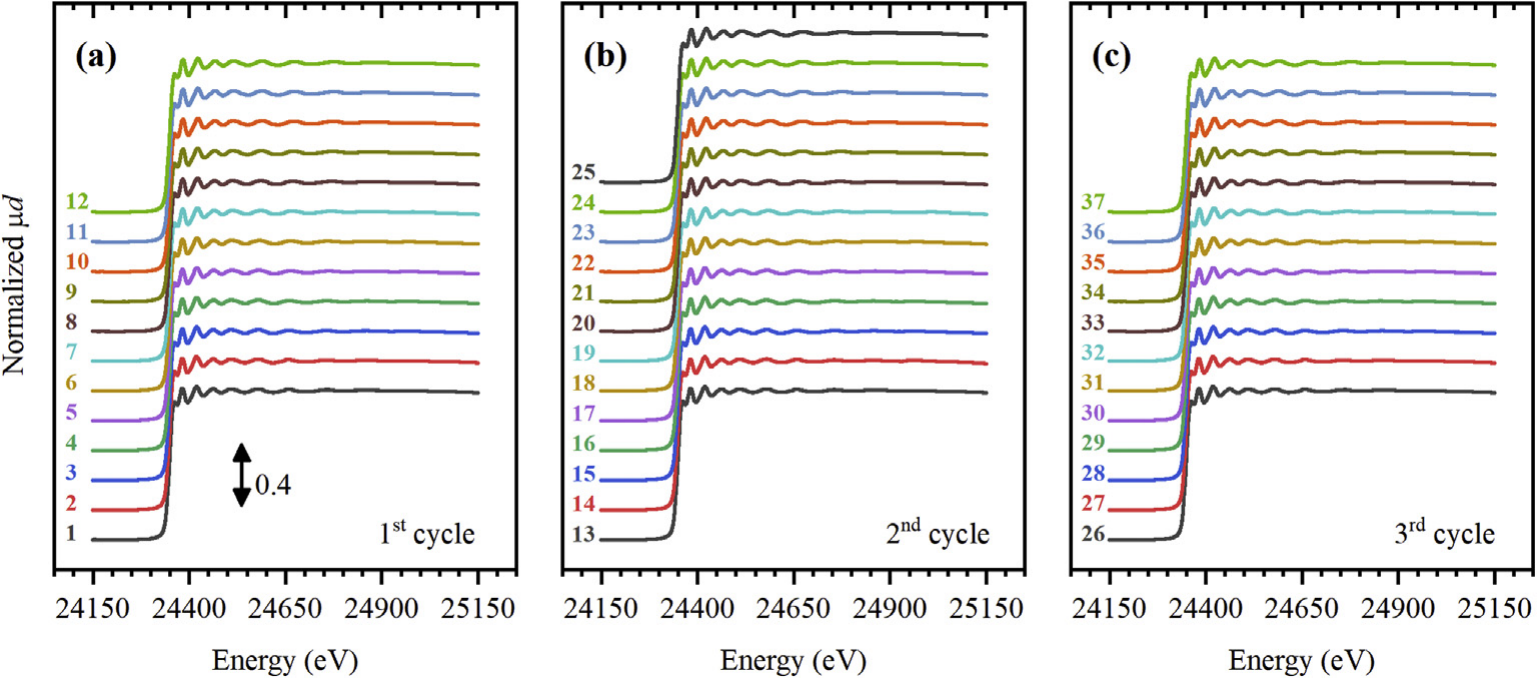
Normalized EXAFS spectra collected during the first (a), second (b) and third (c) ethylene hydrogenation cycles.

**Fig. 2:**
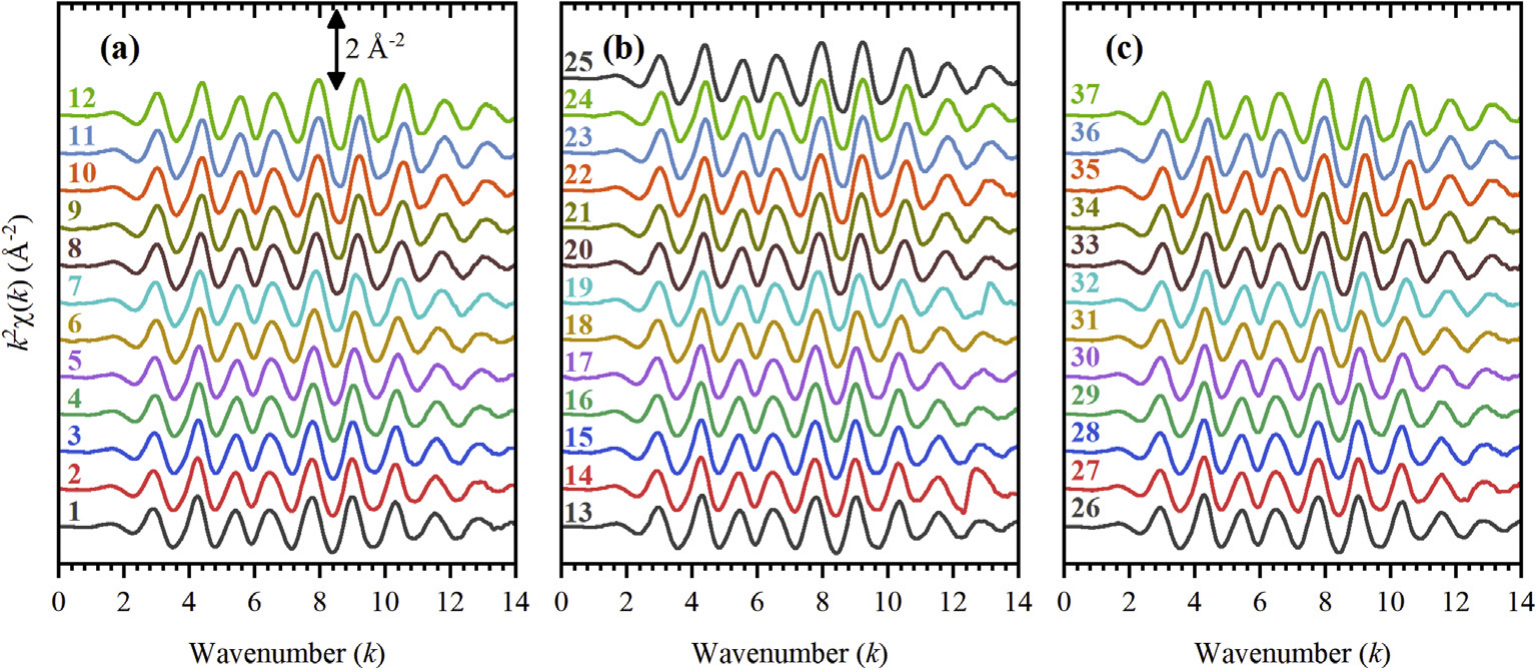
*k*^2^-weighted oscillatory EXAFS functions χ(*k*) of the spectra collected during the first (a), second (b) and third (c) ethylene hydrogenation cycles.

**Fig. 3:**
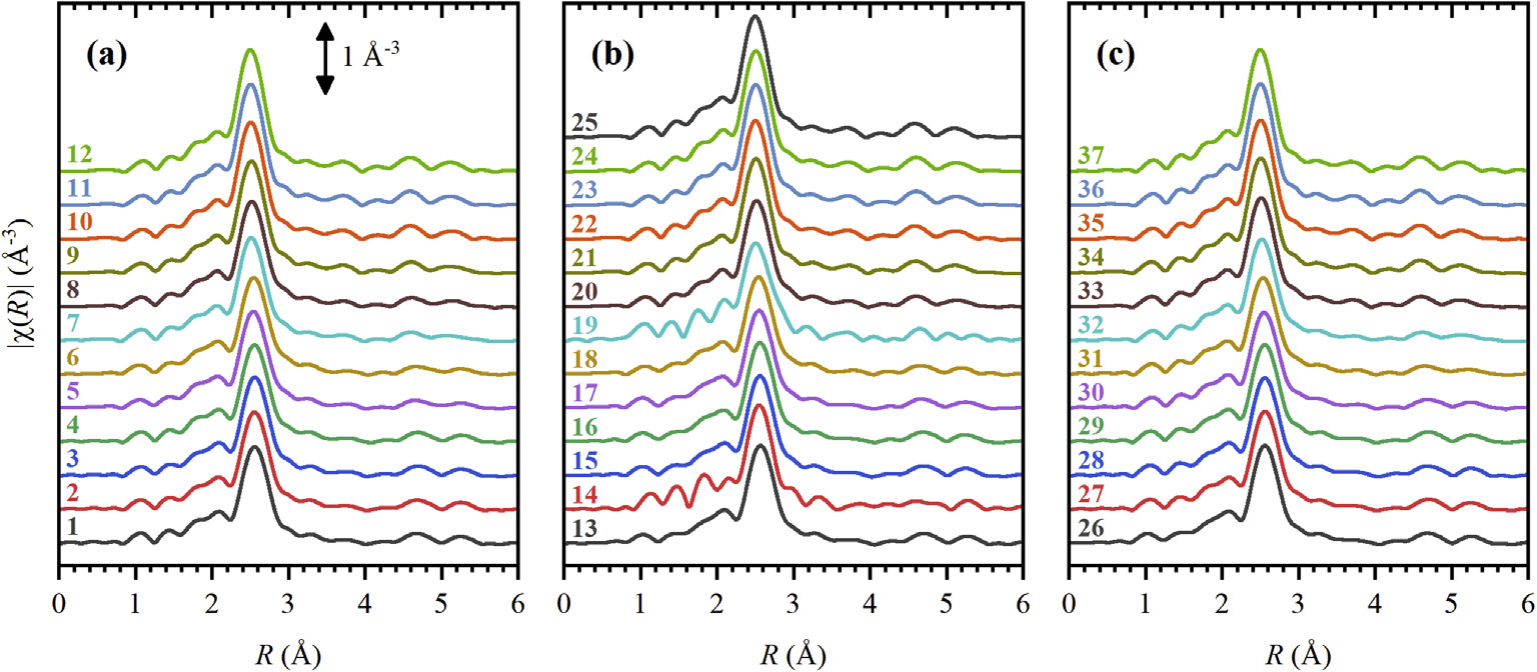
Amplitudes of the *k*^2^-weighted Fourier-transformed EXAFS data, χ(*R*), of the spectra collected during the first (a), second (b) and third (c) ethylene hydrogenation cycles.

**Fig. 4:**
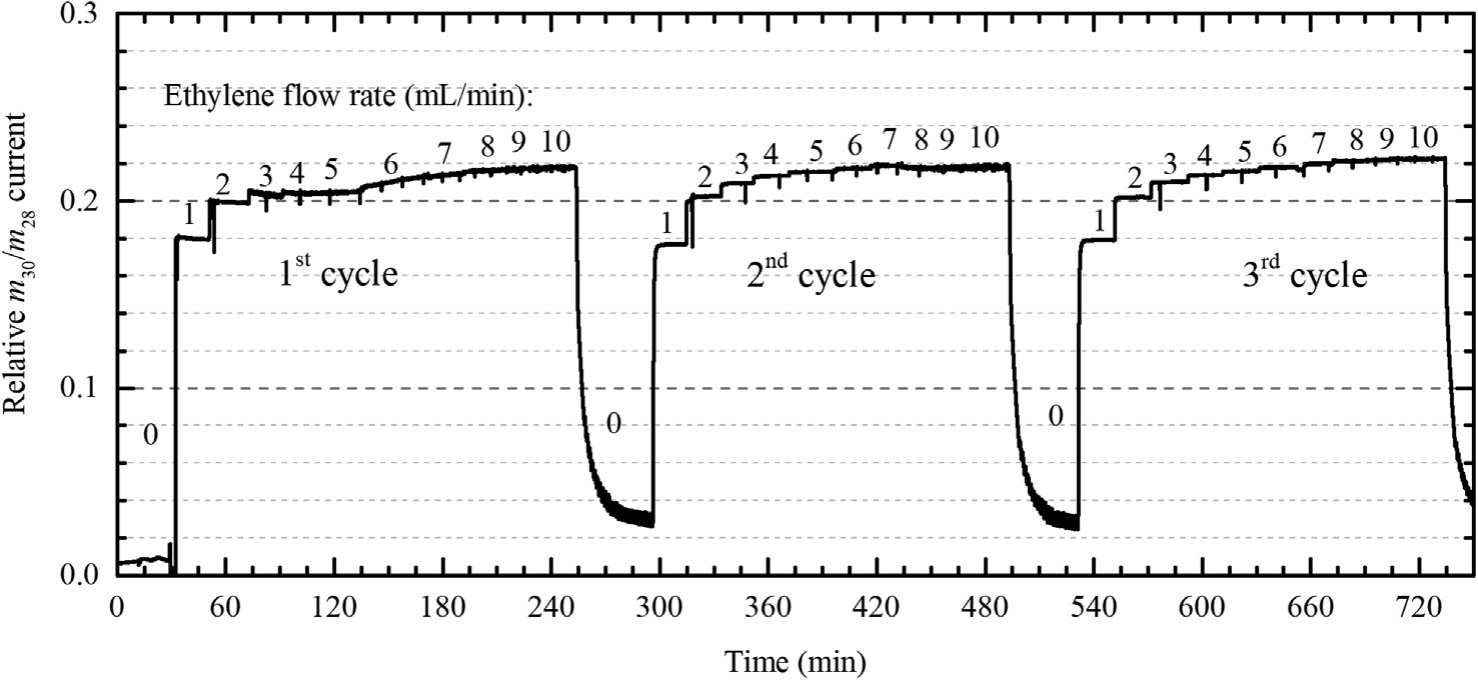
The relative fraction of the MS-detected signals for *m*/*Z* = 30 and *m*/*Z* = 28 as a function of time. The defined ethylene flow rate at different time points are specified in the graph.

The dataset consists of 37 different Pd *K*-edge X-ray absorption spectra collected during ethylene hydrogenation reaction under variable ethylene-to-hydrogen ratio [1]. The spectra are presented in the three forms. First, the initial EXAFS spectra after normalization are presented in Fig. 1. The background subtracted *k*^2^-weighted oscillatory EXAFS functions χ(*k*) are reported in Fig. 2. The *k*^2^-weighted Fourier-transformed χ(*R*) functions are shown in Fig. 3. Normalized EXAFS spectra, χ(*k*) functions, and χ(*R*) Fourier-transforms are listed in the files “muE.dat”, “chiK.dat”, and “chiR.dat”, respectively. The correspondence between the spectrum number and the adopted helium, hydrogen and ethylene flows is given in the Table 1. The time evolution of the ratio of *m*/*Z* signals of 30 and 28, registered by online mass spectrometer, is reported in Fig. 4, the corresponding data are listed in the file MS.dat.

**Table 1:** The correspondence between the spectrum number and the adopted helium, hydrogen and ethylene flows.

1st cycle				2^nd^ cycle				3^rd^ cycle			
#	Flow rate (mL/min)			#	Flow rate (mL/min)			#	Flow rate (mL/min)		
	He	H_2_	C_2_H_4_		He	H_2_	C_2_H_4_		He	H_2_	C_2_H_4_
1	35	15	0	13	35	15	0	26	35	15	0
2	34	15	1	14	35	15	0	27	35	15	0
3	33	15	2	15	34	15	1	28	34	15	1
4	32	15	3	16	33	15	2	29	33	15	2
5	31	15	4	17	32	15	3	30	32	15	3
6	30	15	5	18	31	15	4	31	31	15	4
7	29	15	6	19	30	15	5	32	30	15	5
8	29	15	6	20	29	15	6	33	29	15	6
9	28	15	7	21	28	15	7	34	28	15	7
10	27	15	8	22	27	15	8	35	27	15	8
11	26	15	9	23	27	15	8	36	26	15	9
12	25	15	10	24	26	15	9	37	25	15	10
				25	25	15	10

## 2 Experimental design, materials, and methods

**Sample.** The sample for which all the data were collected is an industrial 5 wt% Pd/C catalyst provided by Chimet S.p.A. The catalyst consists of the palladium nanoparticles supported on the activated wood-based carbon. The average nanoparticle size is 2.6 nm with narrow particle size distribution. Detailed characterization of both the catalyst and the support itself have been reported in a number of works [2], [3], [4], [5], [6], [7], [8], [9], [10], [11], [12], [13].

**EXAFS data collection.** Experimental Pd *K*-edge EXAFS spectra were measured at BM26A beamline [14], [15] of European Synchrotron Radiation Facility (ESRF, Grenoble, France). The X-ray beam was monochromatized by Si(111) double-crystal monochromator and harmonic rejection was performed by Pt coated mirrors. The catalyst was loaded into an *in situ* cell with build-in heater [16]. Two additional stainless steel washers were inserted to increase the thickness of the sample, which resulted in the absorption step Δ*μx* = 0.35. The spectra were collected in transmission mode using ionization chambers. Palladium foil was used to calibrate the energy. The energy range for the spectra was set from 24.15 to 25.15 eV, which corresponds to *k*_max_ around 14.4 Å^−1^ (see Fig. 2). The energy step in the pre-edge region was set to 5 eV with acquisition time of 1.5 s, step in the XANES region was 1 eV with 1.5 s acquisition, and the step in the EXAFS region was defined to obtain a uniform 0.04 Å^−1^ step in the *k*-space. The time per point in the EXAFS region was increasing quadratically from 1.5 to 2.5 s.

**Catalytic reaction.** The cell with the sample was connected to the gas line of BM26A [17]. To avoid overpressure when flowing the gas mixture, the sample was sieved to 100–150 μm before being loaded into the cell. During the catalytic reaction, the pressure was monitored by Bourdon tube pressure gauge. The gas line was equipped with remotely controlled Bronkhorst mass flow controllers (MFCs) and electrovalves. All MFCs were calibrated using Agilent flow meter. Before starting the catalytic reaction, the sample was reduced in hydrogen at 125 °C for 30 minutes. The catalytic hydrogenation of ethylene to ethane was tested by stepwise increasing of the ethylene-to-hydrogen ratio. Helium was used as the carrier gas. The exact flow rates of the three gases are reported in Table 1. Because of the fact that the ethylene-to-hydrogen ratio was changed three times from 0 to 10/15, we virtually divided the whole set of data into three respective parts, which are referred to as 1st cycle, 2nd cycle and 3rd cycle. The products in the gas phase after the sample were monitored by online mass spectrometer manufactured by Pfeiffer. To quantitatively determine the ratio between ethylene and ethane in the output gas mixture, we used the ratio of *m*/*Z* signals 30 and 28. This ratio is changing from 0 for pure ethylene to ca. 0.22 for pure ethane.

**EXAFS data processing.** EXAFS data was processed in a standard way using Demeter package [18]. At the first step, a linear extrapolation of the pre-edge region was used to construct the pre-edge lines. After subtraction of the pre-edge lines the spectra were normalized by post-edge lines, representing second order polynomials. In this form, the spectra are presented in the Fig. 1, and are listed in the Supplementary materials. Then, the smooth background lines were subtracted from the spectra to extract the oscillatory part of EXAFS spectra [19]. To recalculate the incident photon energy *E* to the photoelectron wavenumber *k*, the energy of the edge *E*_0_ was determined as the maximum of the first derivative for each spectrum. To enhance the oscillation at higher *k*-values, the oscillatory functions were multiplied by *k*^2^. These *k*^2^-weighted χ(*k*)-functions are reported in Fig. 2 and are listed in the Supplementary materials. Finally, the *k*^2^-weighted χ(*k*)-functions were Fourier-transformed applying the *k*-window from 3 to 14 Å^−1^. The amplitudes of χ(*k*) are shown in Fig. 3 and are listed in the Supplementary materials. For the analysis presented in the paper [1], the lower boundary of the *k*-window was increased to 4 Å^−1^ to reduce the contribution of the backscattering from carbon atoms of the support [8], [20] and of the palladium carbide phase [21]. The upper boundary was lowered to 12 Å^−1^ to avoid possible effect of the noise at high *k*-values.
