# Supplementary material for: Operando X-ray absorption spectra and mass spectrometry data during hydrogenation of ethylene over palladium nanoparticles
Source: Data Brief. 2019 Apr 26;24:103954. doi: 10.1016/j.dib.2019.103954 (PMC6515128; doi:10.1016/j.dib.2019.103954)
Supplement: Supplementary file 1 — Multimedia component 1 [file mmc1.pdf]

Conflict of Interest and Authorship Conformation Form

Please check the following as appropriate:

- ○ All authors have participated in (a) conception and design, or analysis and interpretation of the data; (b) drafting the article or revising it critically for important intellectual content; and (c) approval of the final version.

- ○ This manuscript has not been submitted to, nor is under review at, another journal or other publishing venue.

- ○ The authors have no affiliation with any organization with a direct or indirect financial interest in the subject matter discussed in the manuscript

- ○ The following authors have affiliations with organizations with direct or indirect financial interest in the subject matter discussed in the manuscript:

Aram L. Bugaev	Southern Federal University, Russia
Alexander A. Guda	Southern Federal University, Russia
Ilia A. Pankin	Southern Federal University, Russia
Elena Groppo	University of Turin, Italy
Riccardo Pellegrini	Chimet S.p.A., Italy
Alessandro Longo	ESRF, France
Alexander V. Soldatov	Southern Federal University, Russia
Carlo Lamberti	University of Turin, Italy

I, Aram Bugaev, sign this form as the corresponding author and on behalf of all co-authors.
